# Complete Genome Sequence of *Brachybacterium* sp. Strain SGAir0954, Isolated from Singapore Air

**DOI:** 10.1128/MRA.00619-19

**Published:** 2019-08-08

**Authors:** Phu Pwint Thin Hlaing, Ana Carolina M. Junqueira, Akira Uchida, Rikky W. Purbojati, James N. I. Houghton, Caroline Chénard, Anthony Wong, Megan E. Clare, Kavita K. Kushwaha, Alexander Putra, Carmon Kee, Nicolas E. Gaultier, Balakrishnan N. V. Premkrishnan, Cassie E. Heinle, Serene B. Y. Lim, Vineeth Kodengil Vettah, Daniela I. Drautz-Moses, Stephan C. Schuster

**Affiliations:** a Singapore Centre for Environmental Life Sciences Engineering, Nanyang Technological University, Singapore; b Departmento de Genética, Instituto de Biologia, Universidade Federal do Rio de Janeiro, Rio de Janeiro, Brazil; Indiana University, Bloomington

## Abstract

*Brachybacterium* sp. strain SGAir0954 was isolated from tropical air collected in Singapore, and its genome was sequenced and assembled using long reads generated by single-molecule real-time (SMRT) sequencing. The complete genome has a size of 3.41 Mb and consists of 2,955 protein coding genes, 50 tRNAs, and 9 rRNAs.

## ANNOUNCEMENT

The genus Brachybacterium was established in 1988 and belongs to the family Dermabacteraceae (1). *Brachybacterium* species are Gram-positive bacteria (2) that vary in shape and exhibit a rod-coccus cycle (1). Species of this genus are ubiquitous and were previously isolated from various sources, such as dogs, laboratory mice, insects, reptiles, fermented foods, poultry deep litter, feces, and environmental samples (2–4). A recent case report documented a *Brachybacterium* sp. as the causative pathogen of bloodstream infection in a human (2); thus, in-depth analysis at the species level of this genus will be beneficial to clarify clinical characteristics of *Brachybacterium* spp.

Strain SGAir0954 was isolated from an air sample collected in Singapore (1.346 N, 103.680 E) using an SASS 3100 dry air sampler (Research International, USA). The filter from the sampler was washed with phosphate-buffered saline (Thermo Fisher Scientific, Singapore) containing 0.1% Triton X-100 (Sigma-Aldrich, Singapore). Particles suspended in the buffer were spread onto Todd-Hewitt agar (Sigma-Aldrich, Singapore) and incubated at 30°C overnight. A clonal culture was obtained by repeated streaking onto new plates of the same type. For DNA extraction, the bacterial colony was grown in lysogeny broth (BD, USA) at 30°C overnight while shaking at 150 rpm. Genomic DNA was purified using the Wizard genomic DNA purification kit (Promega, USA) per the manufacturer’s protocol. The sequencing library was prepared with the SMRTbell template prep kit 1.0 (Pacific Biosciences, USA), and subsequent single-molecule real-time (SMRT) sequencing was performed on the Pacific Biosciences RS II platform. Default parameters were used for all software unless otherwise stated.

A total of 43,042 long reads (*N*_50_ value, 11,181 bp) were generated by SMRT sequencing and used for *de novo* assembly with the Hierarchical Genome Assembly Process (HGAP) version 3 (5) of the PacBio SMRT Analysis 2.3.0 package. Quality control of reads was performed using the PreAssembler Filter version 1 protocol from HGAP, and genome quality was improved by polishing with Quiver (5). The polished assembly was then circularized and reoriented with Circlator 1.1.4 (6). The region of high-similarity overlap was identified, producing a single circular chromosomal contig of 3,410,111 bp (71.8-fold coverage) with a mean G+C content of 73.0%.

Taxonomic assignment was carried out using the average nucleotide identity (ANI) method and 16S rRNA identification, resulting in assignment to the *Brachybacterium* genus. ANI analysis conducted with Microbial Species Identifier (MiSI) (7) was run using ANICalculator with default parameters against a database of 6,387 bacterial RefSeq genomes created using text filter for “type, synonym type, proxytype” and subsequently “getorf -find 3.” This resulted in 82.3% identity with Brachybacterium squillarum M-6-3, with an alignment fraction value of 0.26. The 16S rRNA analysis using Barrnap version 0.7 (8) and BLASTn (9) was run against the SILVA database (10) and resulted in a 100% identity with *Brachybacterium rhamnosum*. As the ANI result is below the threshold for species-level identification, the isolate was assigned to the genus *Brachybacterium* based on the combined ANI and 16S sequence similarity.

The genome was annotated using NCBI’s Prokaryotic Genome Annotation Pipeline (PGAP) version 4.4 (11). The genome was predicted to consist of a total of 3,078 genes, including 2,955 protein-coding genes, 9 rRNA genes (5S, 16S, and 23S), 50 tRNA genes, 3 noncoding RNA genes, and an additional 61 pseudogenes. Using Rapid Annotations using Subsystems Technology (RAST) (12–14) with the ClassicRAST annotation scheme with the “fix frameshift” option set to “yes,” functional annotation revealed that 51 genes were associated with virulence, disease, and defense, which indicates a moderate virulence for this bacterium. Only two genes were found to be associated with dormancy and sporulation, which suggests that *Brachybacterium* spp. may not have an obvious long-term survival mechanism to dominate or outcompete other bacteria.

### Data availability.

The genome sequence of *Brachybacterium* sp. strain SGAir0954 was deposited in the DDBJ/EMBL/GenBank databases under accession number CP027295. Raw data were submitted to the SRA database under the accession number SRR8894409.
